# Interleukin-4 during post-exercise recovery negatively correlates with the production of phagocyte-generated oxidants

**DOI:** 10.3389/fphys.2023.1186296

**Published:** 2023-12-12

**Authors:** Adam Chmielecki, Krzysztof Bortnik, Szymon Galczynski, Karolina Kopacz, Gianluca Padula, Hanna Jerczynska, Robert Stawski, Dariusz Nowak

**Affiliations:** ^1^ Sports Centre, Medical University of Lodz, Łódź, Poland; ^2^ Academic Laboratory of Movement and Human Physical Performance “DynamoLab”, Medical University of Lodz, Łódź, Poland; ^3^ Central Scientific Laboratory, Medical University of Lodz, Łódź, Poland; ^4^ Department of Clinical Physiology, Medical University of Lodz, Łódź, Poland

**Keywords:** exhaustive exercise, interleukin-4, pro-inflammatory cytokines, phagocytes, whole blood luminescence, reactive oxygen species, ROS, granulocytes

## Abstract

Exhaustive run induced a biphasic oxidative response of circulating phagocytes in 16 amateur sportsmen. The first phase involved an increment just after exercise of enhanced whole blood chemiluminescence normalized per phagocyte count, whereas in the second phase a decrement from 1 h post-exercise and ongoing till 24 h. We tested whether plasma Interleukin IL-4, IL-8, IL-10 and Tumor Necrosis Factor α concentrations change in response to exhaustive run and whether there are associations between their levels and delta resting. Moreover, IL-8 and IL-10 significantly increased immediately post-exercise and after 1 h, but later normalized. Tumor necrosis factor α rose by 1.1-times only just after exercise. However, none of these cytokines showed any correlation with the investigated chemiluminescence. Exercise did not alter plasma concentrations of IL-4. However, pre-exercise IL-4 negatively correlated with measured luminescence just after exercise (*ρ* = −0.54, *p* < 0.05), and also tended to be negatively associated with decrements of the second phase at 1 h post-exercise *ρ* = −0.45, *p* = 0.08. It is suggested that plasma IL-4, by a negative association with blood phagocytes oxidants production, could be involved in the maintenance of proper balance between oxidants and anti-oxidants during strenuous exercise and post-exercise recovery.

## 1 Introduction

Strenuous exercise was reported to induce numerous immuno-metabolic responses in well-trained and untrained subjects (Nieman and Pence, 2020; Padilha et al., 2021). These include, for instance, the release of various cytokines (e.g., IL-6, IL-8, IL-10), an increase in intramuscular activities of antioxidant enzymes (superoxide dismutase, catalase, glutathione peroxidase), changes in the number and activity of circulating neutrophils, monocytes, dendritic cells, T cells, and natural killer cells, as well as mitochondrial biogenesis and alterations in gut microbiome (Walsh et al., 2011; Nieman and Pence, 2020; Padilha et al., 2021). Moreover, numerous circulating oxylipins generated during exercise may alter the function of immune system cells (Nieman and Pence, 2020). Although these responses, induced by a single bout of exercise, are transient, properly selected, regular lifelong exercise can delay aging-related immunosenescence (Padilha et al., 2021). On the other hand, prolonged training overload may induce a persistent inflammatory response and oxidative stress, which are contributors to the development of overtraining syndrome (Tanskanen et al., 2010; Cheng et al., 2020). Recently, we found that exhaustive treadmill exercise at a speed corresponding to 70% of personal VO_2_ max caused an increase in resting (spontaneous) production of reactive oxygen species (ROS) by circulating polymorphonuclear leukocytes (PMNs), evaluated by luminol enhanced whole blood chemiluminescence (LBCL) (Chmielecki et al., 2022). This change was observed for both absolute resting chemiluminescence (a-rLBCL—light emission generated by 3 µL of the assayed blood sample) and for chemiluminescence calculated per 103 phagocytes present in the assayed blood sample (rLBCL). However, exercise-induced increase in ROS production was transient and absolute resting chemiluminescence at 1 h post-exercise did not differ from the pre-exercise baseline values. Moreover, resting chemiluminescence s after 1 h from the end of the bout was about 2-times lower than pre-exercise values, and this inhibition persisted till 24 h post-exercise (Chmielecki et al., 2022). Analogous observation, in similar conditions has reported also Yamada et al. (2000); Quindry et al. (2003). We supposed that this increase immediately after exercise in a resting chemiluminescence and rLBCL was related to exercise-induced NETosis (Chmielecki et al., 2022), which is a program for formation of neutrophil extracellular traps (NETs). It is a unique form of cell death that is characterized by the release of decondensed chromatin and other granular contents to the extracellular space. Noteworhty NETosis is offenly accompanied by ROS production (Vorobjeva and Chernyak, 2020) and had similar kinetics (Velders et al., 2014). We also postulated that biphasic changes of resting chemiluminescence (rise just after exercise and then significant suppression) could be the consequence of a parallel increase in circulating anti-inflammatory IL-10 (Chmielecki et al., 2022). IL-10 was reported to rise in response to exercise (Cabral-Santos et al., 2015; Cabral-Santos et al., 2019) and *in vitro* inhibited respiratory burst of PMNs after stimulation with various agonists (Chaves et al., 1996; Capsoni et al., 1997; Dang et al., 2006). Nevertheless, PMNs during and after the exhaustive treadmill run are exposed to a variety of pro- and anti-inflammatory cytokines as TNF-α and IL-8, IL-4, for example,. The first two previously listed are able to prime and stimulate ROS generation from PMNs (You et al., 1991; Baggiolini and Clark-Lewis, 1992; Gougerot-Podicalo et al., 1996; Decleva et al., 2002), while IL-4 was reported to inhibit PMA (phorbol 12-myristate 13-acetate)-induced neutrophil extracellular traps (NETs) formation *in vitro* (Impellizzieri et al., 2019). Having stored plasma specimens obtained from our previous study (Chmielecki et al., 2022), we decided to measure concentrations of these four cytokines (IL-10, TNF-α, IL-8 and IL-4). Hence we hypothesized: (1)—there is an effect of exhaustive exercise on circulating cytokins within 24 h from the end of exercise, and (2)—there is possible associations between circulating cytokins and exercise-induced changes in ROS production by blood PMNs.

## 2 Materials and methods

### 2.1 Studied group

The studied group of 18 average trained non-smoking, healthy men (10 soccer players and 8 powerlifters). The inclusion/exclusion criteria and the study protocol were the same as in our previous report (Chmielecki et al., 2022). Volunteers had mean age 22 ± 2 years, mean body mass 80 ± 12 kg, mean body mass index 24.3 ± 2.6 kg/m^2^, and a mean maximal oxygen consumption 49 ± 5 mL/kg.min^−1^ (Supplementary Tables S1, S2). There were no significant differences between soccer players and powerlifters in respect to the afore-mentioned variables probably due to the short training experience. Exclusion criteria were as follows: presence of contraindications to exhaustive treadmill exercise, presence of any injuries which may limit exercise performance, current cigarette smoking, alcohol and illicit drug abuse, use of any vitamins, food supplements, antioxidants or any systemic pharmacological treatment or history of acute infectious or inflammatory diseases within 3 months prior to the study.

### 2.2 Study protocol

Briefly, the study had a cross over design and consisted of four visits on the 0th, 7th, 14th, and 28th day of observation (Chmielecki et al., 2022) (Table 1). The first two visits (0th and 7th) were focused on volunteers eligibility criteria, obtaining informed consent, collecting demographic data, exclusion of contraindications to exercise stress, assessment of VO_2_max, and randomization with assignment to arm 1 or 2. During the third visit (14th), half of the randomly selected participants (arm 1, *n* = 9) performed a treadmill run to volitional exhaustion at a speed corresponding to 70% of their personal VO_2_max. Subjects were only allowed to drink mineral water at will during the treadmill run, and pre- and post-exercise heart rate, arterial blood pressure, and body mass were measured. Venous blood samples (15 mL) were collected six times into Becton Dickinson vacutainer tubes (with EDTA for blood cell count, for resting chemiluminescence and for IL-4, IL-8, IL-10 and TNF-α evaluation) with gel and clot activator for blood chemistry and with sodium oxalate and potassium fluoride for lactate measurement before, just after, and at 1, 3, 5 and 24 h after the exhaustive exercise. Blood chemistry and lactate were determined only in pre- and immediately post-exercise blood samples. EDTA anticoagulated blood samples were centrifuged for 15 min (1000 × g, 4°C) and the obtained plasma was stored at −80°C (up to 5 months), until cytokine measurement. Volunteers allocated to arm 2 (*n* = 9) underwent the same procedures but without the exhaustive treadmill exercise. At the 4th visit (day 28th), subjects from arm 2 performed the exhaustive treadmill run while those from arm 1 were without any physical effort (Table 1). Volunteers nutrition, training load restrictions during this 28 days study, and ambient conditions during the exhaustive treadmill run have been described in detail in our previous work (Chmielecki et al., 2022). This study was conducted according to the Declaration of Helsinki, the protocol was reviewed and approved by The Medical University of Lodz Ethics Committee (RNN/118/17/KE), and all volunteers provided a written informed consent.

**TABLE 1:** Study design and summary of protocol procedures.

Visit number	Day of the study, time	Procedures performed during the visit	
1	0th, 9 a.m.–2 p.m.	Volunteers enrollment (eligibility criteria - inclusion/exclusion criteria), informed consent, instructing the volunteer about all study procedures	
2	7th, 9:30 a.m.	Medical examination, resting ECG, spirometry, arterial blood pressure, treadmill VO_2_max assessment, randomization	
3	14th, 9:30 a.m.	Medical examination, resting ECG, arterial blood pressure, blood collections—before, just after, and at 1, 3, 5 and 24 h post-exercise	
		Arm 1	Arm 2
		Treadmill (constant inclination of 1.5%) run to volitional exhaustion at a speed corresponding to 70% of personal VO_2_max	1 h resting instead of treadmill run
4	28th, 9:30 a.m.	Medical examination, resting ECG, arterial blood pressure, blood collections—before, just after, and at 1, 3, 5 and 24 h post-exercise	
		1 h resting instead of treadmill run	Treadmill (constant inclination of 1.5%) run to volitional exhaustion at speed corresponding to 70% of personal VO_2_ max

a.m.—Ante Meridiem, ECG-electrocardiography, VO_2_max–maximal oxygen consumption.

Volitional exhaustion was defined as the volunteers’ inability to maintain the required exercise intensity or their will to stop the treadmill run, despite a strong encouragement to continue by the testing staff. When symptoms of volitional exhaustion appeared, the exercise test was terminated. Mean run distance to exhaustion was 13.9 ± 5.1 km, run time 76 ± 26 min, heart rate at the end of the run 167 ± 12 beats/min, representing 84% ± 7% of maximal heart rate, and 1.2 ± 0.5 kg loss of body mass.

### 2.3 Description of VO_2_ max and execution of exhaustive treadmill run

The exhausting treadmill run and the VO_2_ max measurement (continuous incremental maximal exercise test) were carried out in accordance with the same protocol, using the same technical tools, and under the same circumstances as previously mentioned (Chmielecki et al., 2022). Briefly, volunteers began running on a treadmill (continuous incline of 1.5%) at a pace of 7 km/h; this speed was increased by 1.5 km/h every 3 minutes until voluntary exhaustion. To estimate VO_2_ max, three conditions required to be met: (A) a plateau in oxygen consumption despite an increase in running speed; (B) a respiratory exchange ratio more than 1.10; and (C) a peak heart rate greater than 90% of the age-predicted maximum (220—age). The strenuous exercise was a treadmill run at a speed equal to 70% of the body’s measured maximum heart rate.

### 2.4 Measurement of the luminol-enhanced whole blood chemiluminescence

Resting luminol enhanced whole blood chemiluminescence, as a measure of *ex vivo* spontaneous ROS production by circulating phagocytes, was carried out according to the protocol described by Kukovetz et al. (1997) with our modifications (Bialasiewicz et al., 2014; Bialasiewicz et al., 2018) described in detail in our previous study (Chmielecki et al., 2022). The luminol solution was prepared by dissolving 25 mg luminol in 90 mL (0.1 mol/L) Na2HPO4, then the pH was adjusted to 7.4 with 1 mol/L HCl, and the volume was made up to 100 mL with distilled water. After immediate filtration (0.2 mm Millipore filter), it was stored at 4°C in the dark for no longer than 2 weeks. The mixture solution was prepared just before the blood chemiluminescence assay by adding 1 mL of Ringer’s solution (155.7 mmol/L NaCl, 5.36 mmol/L KCl, 1.78 mmol/L NaHCO3, pH = 7.4), 5 mL of luminol solution, and 0.2 mL of 277.5 mmol/L glucose solution to 3.6 mL distilled water. Briefly, blood samples were initially diluted with a mixture solution (30 µL of blood added to 1000 µL of mixture solution). Then, 103 µL of diluted blood was added to a tube (Lumi Vial Tube, 5 mL, 12 × 75 mm, Berthold Technologies, Bad Wildbad, Germany) containing 797 µL of mixture solution (which resulted in a final blood dilution of 300 times), placed in a multitube luminometer (AutoLumat Plus LB 953, Berthold, Germany) and incubated for 15 min at 37°C in the dark. Then, 100 µL of the control solution (0.9% NaCl with addition of 1% v/v DMSO) for measurement of absolute resting chemiluminescence was injected by an automatic dispenser, and, after 7 s, the total light emission was automatically measured for 120 s. Individual results (given in relative light units—RLU) were obtained as the means of triplicate experiments. To exclude the possible fluctuations of the background noise signal and its effect on chemiluminescence, the light emission from samples containing only 900 µL of mixture solution was measured before and after each series of six studied samples, then the mean background noise signal was subtracted from the corresponding individual results of absolute resting chemiluminescence. Results of resting chemiluminescence were expressed in two ways: (A) as absolute light emission generated by 3 µL of assayed blood sample absolute resting chemiluminescence, and (B) as light emission per 10^3^ phagocytes (granulocytes and monocytes) present in the assayed blood sample resting chemiluminescence.

### 2.5 Measurement of IL-4, IL-8, IL-10 and TNF-α in plasma samples

Concentrations of IL-4, IL-8, IL-10 and TNF-α were simultaneously measured in each plasma sample using a multiplex kit based on the Luminex xMAP technology (Human High Sensitivity Cytokine A Assay; Cat # FCSTM09, R&D Systems, United States). All procedures were strictly executed according to the manufacturer instruction (Magnetic Luminex Performance Assay, www.RnDSystems.com). The results are presented as the mean of two repeats whereas the intra assay CV is parameter automatically analyzed by algorithm attached to the equipment thus results with CV higher than 10% were reapeted. Briefly, four types of magnetic beads pre-coated with antibodies specific for each afore-mentioned analyte (25 µL of diluted Microparticle Cocktail) were added to the wells containing 100 µL of standards, plasma samples or controls, and incubated for 3 h. During this incubation step, analytes of interest from the standard and plasma samples were captured by the appropriate beads. Then, the magnetic beads were washed three times with 100 µL of Wash Buffer and a cocktail of biotinylated antibodies specific for each cytokine (50 µL of diluted Biotin-Antibody Cocktail) was added and incubated for 1 h. Afterwards, all unbound biotinylated antibodies were washed away and 50 µL of streptavidin-phycoerythrin (PE) conjugate was added to detect biotinylated antibodies on the surface of each bead (30 min incubation). At the end, the unbound streptavidin-PE conjugate was removed, the magnetic beads were resuspended in 100 µL of Wash Buffer, and a fluorescence signal proportional to the amount of analyte bound was read on a Luminex^®^ MAGPIX^®^ analyzer (Luminex, United States). All incubations were performed at room temperature and on a shaker at 800 rpm. In the analyzer, the magnetic beads captured in a monolayer were illuminated with two spectrally different diodes (LEDs). One (red) to excite the dyes inside the beads and identify each cytokine, the other (green) to excite PE and measure the amount of analyte bound to the bead. All results were analyzed with Belysa software 1.1.0 (Millipore Sigma, Burlington, MA, United States) and the protein concentration (pg/mL) was determined by interpolation from the standard curve against a five parameter logistic curve. The standard curve range for IL-4, IL-8, IL-10 and TNF-α were 6.6–6 745 pg/mL, 0.8–3 370 pg/mL, 0.5–2 075 pg/mL and 0.8–3 185 pg/mL, respectively. Due to the accidental loss of two sets of plasma samples, the afore-mentioned evaluations were performed for 16 male athletes. Thus, all results related to cytokines including correlations were obtained from a group of 16 subjects.

### 2.6 Other analyses

Blood chemistry (creatine kinase–CK, aspartate amino-transferase, alanine aminotransferase, lactate, urea, creatinine) was determined in the Diagnostic Laboratory of the Central Clinical Hospital of the Medical University in Lodz. Blood cell count was measured with a Micros Analyzer OT 45 (ABX, Montpellier, France). VO_2_ max was determined by a continuous incremental maximal exercise treadmill test (constant inclination of 1.5%, initial speed of 7 km/h increased every 3 min by 1.5 km/h until volitional exhaustion) as previously described (Stawski et al., 2017; Stawski et al., 2019). Three criteria had to be met to determine the VO_2_ max: (A) a plateau in the O2 consumption despite an increase in running velocity, (B) a respiratory exchange ratio higher than 1.10, and (C) a peak heart rate higher than 90% of the age-predicted maximum (220-age) (Tanaka et al., 2001).

### 2.7 Statistical analysis

Results were expressed as means (SD) and medians (interquartile range). Analysis of variance (ANOVA) for repeated observations (parametric test) or Friedman’s ANOVA (nonparametric test) was applied for the assessment of changes in variables over time (pre-exercise, just after, and at 1, 3, 5 and 24 h post-exercise) depending on the data distribution tested with Shapiro-Wilk’s W test. When ANOVA was statistically significant, *post hoc* analyses were done with Scheffe’s test or *post hoc* analysis for Friedman’s ANOVA (multiple comparisons at two different time-points). Individual changes in absolute resting chemiluminescence and resting chemiluminescence from the baseline at given time-point after the exhaustive run (Δa-rLBCL, ΔrLBCL) were calculated by subtracting the pre-exercise value from the appropriate post-exercise value. The relationships between individual changes (increase or decrease) in rLBCL or a-rLBCL and corresponding concentrations of circulating cytokines (IL-4, IL-8, IL-10 and TNF-α) were analyzed with Spearman’s rank correlation coefficient test (Spearman’s *ρ*). Other associations (e.g., between cytokine concentrations and parameters of treadmill run, markers of muscle damage and metabolic response to exercise) were determined in the same way. A *p* value < 0.05 was considered significant. Despite the fact that number of participants was low, our analysis confirmed that the number of participants was sufficient for all analytes besides IL-10. Thus, individual data have been added in the Supplementary File. All calculations and analyses were performed with Dell Statistica (data analysis software system), version 13 (Dell Inc. 2016).

## 3 Results

### 3.1 Muscle damage and metabolic response to exhaustive exercise

This part has been accurately described in our previous study (Chmielecki et al., 2022). Among the six studied markers, aspartate aminotransferase, alanine aminotransferase, creatine kinase, lactate, creatinine and urea, the last four increased significantly after the exhaustive run. The exercise-induced increase in CK, lactate, creatinine and urea were from 180 ± 59 to 233 ± 56 U/L, from 1.7 ± 0.3 to 3.0 ± 1.1 mmol/L, from 88 ± 9 to 106 ± 16 µmol/L and from 5.7 ± 1.1 to 6.5 ± 1.1 mmol/L (*p* < 0.05), respectively. The number of phagocytes (neutrophils plus monocytes) (×103/µL) in the peripheral blood increased from a pre-exercise baseline value of 3.25 ± 1.52 to 5.35 ± 2.49 just after exercise; 6.57 ± 3.39; 8.03 ± 3.78 and 6.45 ± 3.31 at 1, 3, and 5 h post-exercise, respectively, and returned to baseline at 24 h post-exercise (3.12 ± 1.34).

### 3.2 Effect of exercise on plasma concentrations of IL-4, IL-8, IL-10 and TNF-α

**FIGURE 1:**
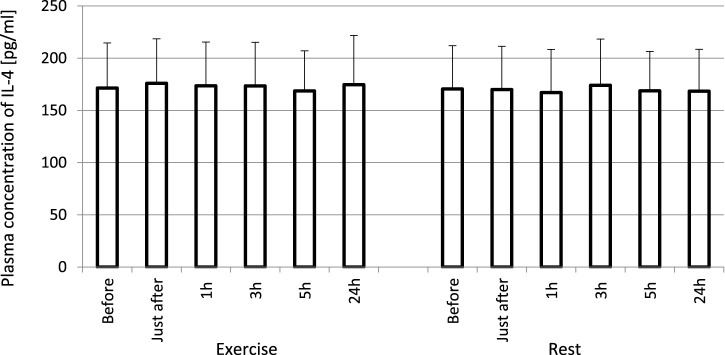
No effect of exhaustive treadmill run on plasma concentrations of IL-4 in amateur athletes. Exercise—subjects performed a treadmill run to volitional exhaustion at a speed corresponding to 70% personal VO_2_ max and blood was collected before, just after, and at 1, 3, 5, and 24 h post-exercise. Rest—control, the same protocol but without any physical activity. Results are expressed as Mean (SD).

**FIGURE 2:**
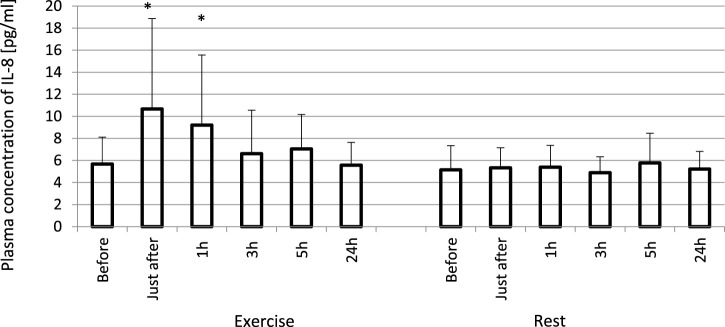
Exhaustive exercise-induced increase in plasma concentration of IL-8 in amateur athletes. *—vs. pre-exercise (before) value, *p* < 0.05. Exercise subjects performed a treadmill run to volitional exhaustion at a speed corresponding to 70% personal VO_2_ max and blood was collected before, just after, and at 1, 3, 5, and 24 h post-exercise. Rest—control, the same protocol but without any physical activity. Results are expressed as Mean (SD).

**FIGURE 3:**
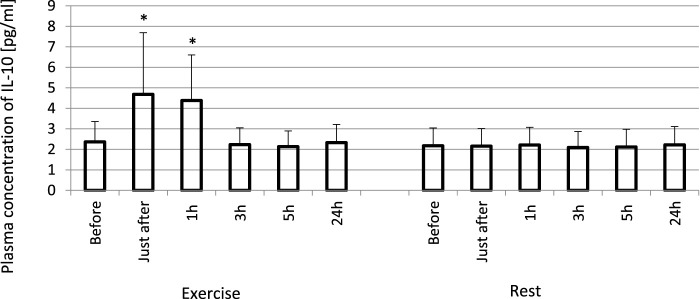
Exhaustive exercise-induced increase in plasma concentration of IL-10 in amateur athletes. *—vs. pre-exercise (before) value, *p* < 0.05. Exercise subjects performed a treadmill run to volitional exhaustion at a speed corresponding to 70% personal VO_2_ max and blood was collected before, just after, and at 1, 3, 5, and 24 h post-exercise. Rest—control, the same protocol but without any physical activity. Results are expressed as Mean (SD).

**FIGURE 4:**
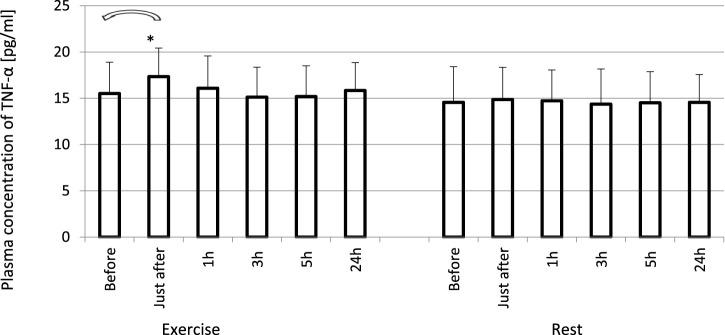
Changes of circulating concentrations of TNF-α in response to an exhaustive treadmill run. *—vs. pre-exercise (before) value, *p* < 0.05. Exercise subjects performed a treadmill run to volitional exhaustion at a speed corresponding to 70% personal VO_2_ max and blood was collected before, just after, and at 1, 3, 5, and 24 h post-exercise. Rest—control, the same protocol but without any physical activity. Results are expressed as Mean (SD).

The exhaustive treadmill run had no effect on circulating concentrations of IL-4 (Figure 1). The post-exercise concentrations ranged from 169 ± 38 (172; 49) pg/mL at 5 h post-exercise to 176 ± 43 (173; 51) pg/mL just after the exercise, and did not differ from pre-exercise ones [171 ± 43(175; 55) pg/mL] as well as to those observed when the exercise was replaced by 1 h resting period (Figure 1). The remaining three cytokines IL-8, IL-10 and TNF-α raised significantly in response to exhaustive run (Figures 2, 3, 4). Mean plasma concentrations of IL-8 observed just after [10.7 ± 8.2 (7.6; 4.1) pg/mL] and at 1 h post-exercise [9.2 ± 6.3 (7.1; 2.9) pg/mL] were 1.9- and 1.6-times higher than those before the bout [5.7 ± 2.4 (5.0 ± 1.8) pg/mL]. Then, the IL-8 level gradually decreased at 24 h post-exercise to a value close to baseline (Figure 2). Similarly behaved IL-10, its concentrations just after [4.7 ± 3.0 (4.5; 3.6) pg/mL] and at 1 h post-exercise [4.4 ± 2.2 (4.0; 3.9) pg/mL] were almost 2-times and 1.8- times higher (*p* < 0.05) than the pre-exercise baseline value [2.4 ± 0.9 (2.5; 1.8) pg/mL], with a following normalization already at 3 h post-exercise (Figure 3). Moderate increase in plasma concentration of TNF-α was noted only just after exercise in comparison to pre-exercise baseline: 17.3 ± 3.1 (16.8 ± 3.6) pg/mL vs. 15.5 ± 3.4 (15.5; 3.9) pg/mL, *p* < 0.05 (Figure 4). No statistically significant IL-8, IL-10 and TNF-α fluctuations were observed in the resting paticipants, i.e., the group without any physical activity (Figures 2, 3, 4) (Supplementary Tables S3–S6).

### 3.3 Effect of exercise on absolute rLBCL (a-rLBCL) and rLBCL expressed as light emission per 10^3^ phagocytes (normalized per phagocyte count)

**FIGURE 5:**
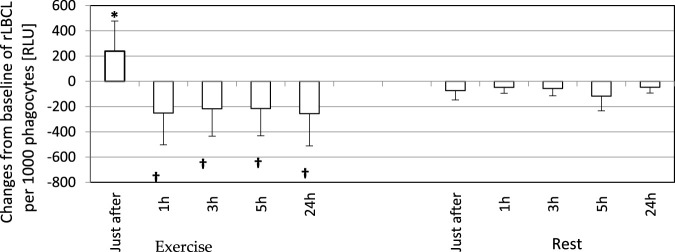
Changes from baseline of rLBCL (light emission per 10^3^ phagocytes) observed just after, and at 1, 3, 5 and 24 h post-exercise. As a baseline served pre-exercise rLBCL. Exercise—eighteen athletes performed a treadmill run to volitional exhaustion at a speed corresponding to 70% personal VO_2_ max and blood was collected before, just after, and at 1, 3, 5, and 24 h post-exercise. Rest - control, the same protocol but without any physical activity. Results are expressed as Mean (SD). *—significant enhancement, †—significant inhibition. The response of rLBCL to an exhaustive run is biphasic: a first phase - increase just after excise; a second phase—decrease at 1 h post-exercise ongoing until 24 h post-exercise.

**FIGURE 6:**
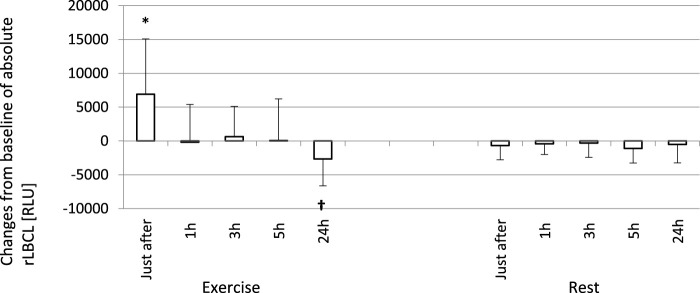
Changes from baseline of absolute rLBCL (a-rLBCL - light emission generated by phagocytes present in 3 µL of assayed blood sample) were observed just after and 1, 3, 5 and 24 h post-exercise. As a baseline served pre-exercise a-rLBCL. Other details as for Figure 5. Results are expressed as Mean (SD). *—significant enhancement, †—significant inhibition. The response of a-rLBCL to an exhaustive run is also biphasic: a first phase - increase just after the exercise, and a second phase - decrease at 24 h post-exercise are separated by a plateau (time points 1, 3, and 5 h post-exercise).

In order to analyze the associations between spontaneous ROS production by circulating phagocytes and plasma concentration of selected cytokines, we calculated rLBCL and a-rLBCL changes from baseline (ΔrLBCL, Δa-rLBCL) over 24 h of post-exercise observations. ΔrLBCL reached 239 ± 398 (193; 495) RLU just after the exercise and −251 ± 467 (−131; 277) RLU, −217 ± 452 (−105; 259) RLU, −216 ± 480 (−118; 196) RLU, −256 ± 432 (−111; 374) RLU at 1, 3, 5 and 24 h post-exercise, respectively (Figure 5), depicting significant enhancement (first time-point) or inhibition (the remaining time-points) of ROS production by exhaustive exercise. In control experiments (rest), ΔrLBCL ranged from −46 ± 222 RLU to −117 ± 237 RLU at 24 h and 5 h post-exercise, illustrating no significant alterations of ROS production in the absence of exhaustive exercise. Δa-rLBCL was 6907 ± 8167 (4356; 2535) RLU and −2673 ± 3965 (−1085; 4154) RLU just after and at 24 h post-exercise representing significant enhancement and inhibition in response to exercise, respectively (Figure 6).

### 3.4 Correlations between selected parameters of exhaustive run and increased post-exercise cytokines

**FIGURE 7:**
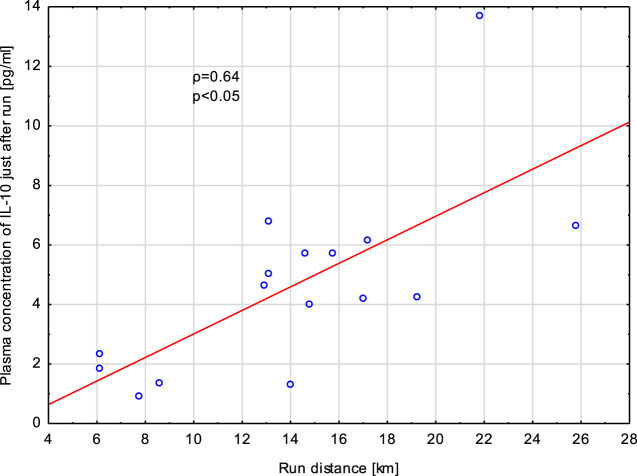
Correlation (Spearman’s ρ) between plasma concentration of IL-10 in samples collected just after exhaustive exercise and run distance. Athletes performed a treadmill run to volitional exhaustion at a speed corresponding to 70% personal VO_2_ max.

Increased concentrations of IL-8 (just after exercise and at 1 h post -exercise) and TNF-α (just after exercise) did not correlate with the run distance, run time, heart rate at the end of run and loss of body mass. However, concentrations of IL-10 in blood specimens collected just after the exercise correlated significantly (*p* < 0,05) with the run distance (*ρ* = 0.62) (Figure 7), run time (*ρ* = 0.57), and loss of body mass (*ρ* = 0.58). Similarly, there were significant positive associations between elevated plasma levels of IL-10 at 1 h post-exercise with run distance and run time (Table 2).

**TABLE 2:** Correlations (Spearman’s *ρ*) between increased post-exercise concentrations of plasma IL-8, IL-10, TNF-α and selected parameters of exhaustive treadmill run.

Parameter	Concentrations of circulating cytokines †				
	IL-8		IL-10		TNF-α
	Just after exercise	1 h post-exercise	Just after exercise	1 h post-exercise	Just after exercise
Run distance	0.35	0.18	0.62*	0.63*	0.07
Run time	0.30	0.11	0.57*	0.57*	0.09
Heart rate at the end of run	0.22	0.43	0.19	0.30	−0.32
Loss of body mass‡	0.45	−0.04	0.58*	0.45	−0.04

^†^ —correlations were calculated only for time-points at which a significant rise of IL-8, IL-10, and TNF-α, was noted.

^‡^ —due to water loss with sweat and evaporation from the surface of airways. Athletes performed a treadmill run to volitional exhaustion at a speed corresponding to 70% personal VO_2_ max. Blood was collected before, just after, and at 1, 3, 5, and 24 h post-exercise. *-p<0.05.

### 3.5 Correlations between exercise-induced increment in luminol enhanced whole blood chemiluminescence and cytokine concentrations

**FIGURE 8:**
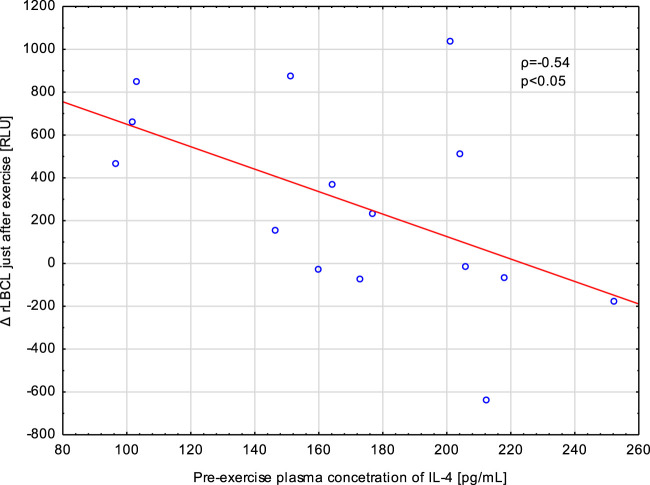
Negative correlation (Spearman’s ρ) between the increment in spontaneous luminol enhanced blood chemiluminescence per 10^3^ phagocytes (ΔrLBCL) just after exercise and pre-exercise plasma concentration of IL-4 in male athletes who performed a treadmill run to volitional exhaustion at a speed corresponding to 70% personal VO_2_ max.

Exhaustive treadmill run increased a-rLBCL and rLBCL of blood samples collected just after the bout. We tested whether pre-exercise or just after the exercise plasma concentrations of four monitored cytokines (IL-4, IL-8, IL-10 and TNF-α) correlated with Δa-rLBCL or ΔrLBCL. TNF-α and IL-8 did not associate either with Δa-rLBCL or with ΔrLBCL (Table 3). There was also no significant association between pre-exercise and just-after-exercise plasma IL-10 levels and Δa-rLBCL. On the other hand, there was a negative correlation between pre-exercise IL-10 and ΔrLBCL (*ρ* = −0.46) that reached borderline significance (*p* = 0.075). Pre-exercise circulating IL-4 negatively correlated (*p* < 0.05) with Δa-rLBCL (*ρ* = −0.51) and ΔrLBCL (*ρ* = −0.54) (Figure 8).

**TABLE 3:** Correlations (Spearman’s *ρ*) between the increment (+Δ) of resting blood chemiluminescence observed just after the exhaustive exercise and plasma cytokine concentrations (IL-4, IL-8, IL-10 and TNF-α).

Plasma cytokine concentrations [pg/mL]	Increment (+Δ) in blood chemiluminescence [RLU] just after the exhaustive treadmill run	
	Δa-rLBCL	ΔrLBCL
IL-4 pre-exercise	−0.51*	−0.54*
IL-4 just after exercise	−0.40	−0.44 ‡
IL-8 pre-exercise	0.07	−0.06
IL-8 just after exercise	0.19	0.06
IL-10 pre-exercise	−0.24	−0.46 †
IL-10 just after exercise	−0.26	−0.31
TNF-α pre-exercise	0.05	0.11
TNF-α just after exercise	0.13	0.23

RLU, relative light units; a-rLBCL, absolute resting luminol enhanced whole blood chemiluminescence; rLBCL, resting luminol enhanced whole blood chemiluminescence per 10^3^ phagocytes, Δa-rLBCL = (a-rLBCL just after exercise)—(pre-exercise a-rLBCL), ΔrLBCL = (rLBCL, just after exercise)—(pre-exercise rLBCL). Blood was collected before (pre-exercise), just after, and at 1, 3, 5, and 24 h post-exercise. *-p<0.05, borderline significance—‡—*p* = 0.099, †—*p* = 0.075.

### 3.6 Correlations between post-exercise decrements (-Δ) in spontaneous luminol enhanced whole blood chemiluminescence and cytokine concentrations

**FIGURE 9:**
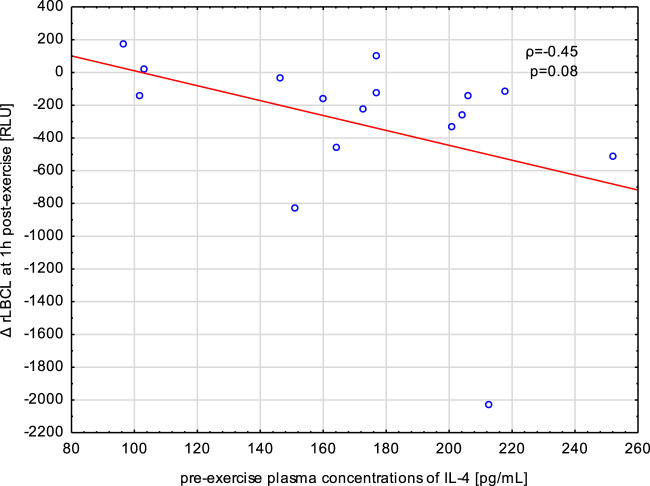
Negative correlation (Spearman’s ρ) between the decrement in spontaneous luminol enhanced whole blood chemiluminescence per 10^3^ phagocytes (ΔrLBCL) at 1 h post-exercise, and pre-exercise plasma concentration of IL-4 in male athletes who performed a treadmill run to volitional exhaustion at a speed corresponding to 70% personal VO_2_ max.

Blood samples collected from male athletes revealed decreased rLBCL at 1, 3, 5, and 24 h post-exercise in comparison to pre-exercise baseline. We analyzed whether circulating concentrations of cytokines (IL-4, IL-8, IL-10 and TNF-α) associated with these decrements in rLBCL. Delat rLBCL (ΔrLBCL) at 1 h post-exercise was correlated with pre-, just-after and 1 h post-exercise concentrations of a given cytokine while ΔrLBCL at 24 h post-exercise was correlated with six sets of 16 individual cytokine levels from 6 time-points what gives in total 96 pairs. For all analyzed pairs (*n* = 96) of ΔrLBCL and cytokine concentration, no significant correlations were found. However, it should be pointed out that for the pair ΔrLBCL and IL-4 (*n* = 16) the Spearman’s *ρ* was always negative and ranged from −0.45 to −0.21. Correlations between ΔrLBCL at 1 h post-exercise and pre- and just-after-exercise IL-4 reached −0.45 (*p* = 0.08) and −0.43 (*p* = 0.09), respectively (Figure 9).

## 4 Discussion

Exhaustive treadmill run resulted in a transient increase in plasma concentrations of three out of four monitored cytokines in amateur sportsmen. Thus, while interleukin IL-8, IL-10 and TNF were increased, IL-4 remained unafected thoughout the 24 h obsevation period. Both IL-8 and IL-10 were elevated at least up to 3 h post-exercise, whereas TNF-α increase was less pronounced and noted only just after the exercise. Only elevated concentrations (just after and at 1 h post-exercise) of IL-10 correlated significantly with exercise bout parameters such as run distance, run time and loss of body mass due to sweat and water evaporation. On the other hand, the increase in IL-8 and TNF-α in response to exercise could be a secondary phenomenon to various immuno-metabolic process activated by exhaustive run. Resting skeletal muscles contain mRNA for all the afore-mentioned cytokines (Peake et al., 2015). Circulating IL-10 and skeletal muscle IL-10 mRNA rose after exercise, while no expression of muscle IL-10 was found (Peake et al., 2015). This indicates that skeletal muscles are not a source of post-exercise increase in plasma IL-10. However, the distinct correlation between increased concentrations of IL-10 and the load of the exhaustive run observed in our study as well as reported by other authors (Cabral-Santos et al., 2019; Suzuki, 2019) suggests that skeletal muscle can generate signals inducing the secretion of IL-10 from other tissues or cells (e.g., Th2 cells) (Kakanis et al., 2014). Furhermore, other cells besides muscle fibers present in skeletal muscles such as macrophages, endothelial cells, satellite cells, fibroblast or pericytes could be involved in the post-exercise IL-10 elevation (Peake et al., 2015). Although TNF-α expression markedly rose in muscles during exercise (Peake et al., 2015), the post-exercise circulating concentrations increased moderately and very transiently. Exercise enhanced expression of IL-8 and IL-10 mRNA in leukocytes, while spontaneous release of these cytokines from leukocytes was decreased (Peake et al., 2015), despite their increase in plasma. Therefore, further studies are necessary to precisely describe mechanisms and sources of exercise-induced changes in plasma cytokine concentrations.

In parallel to transient increase in plasma concentrations of IL-10 and TNF-α, samples of blood collected just after the exercise revealed increased rLBCL that at 1 h post-exercise and later became suppressed in comparison to pre-exercise rLBCL. Surprisingly, there were no significant correlations between ΔrLBCL and plasma concentrations of IL-8, IL-10 and TNF-α just after the exercise. Besides, pre-exercise and just-after-bout concentrations of IL-4 negatively correlated with just-after-exercise ΔrLBCL. It is supposed that changes of PMNs activity including increase in ROS production (Chmielecki et al., 2022), increase in cell-free DNA (Breitbach et al., 2012; Stawski et al., 2017), H3 histone (Stawski et al., 2021) and granular enzymes (e.g., myeloperoxidase, elastase) (Breitbach et al., 2012) in response to vigorous exercise are the consequences of increased NETs formation (Breitbach et al., 2012; Breitbach et al., 2014; Beiter et al., 2015). Moreover, recent studies showed that released cell free DNA in response to exercise almost exclusively originates from neutrophils (Neuberger et al., 2022). IL-4 was reported to inhibit NETs formation *in vitro* by imparing migration of neutrofils, by desensitizing *CXCL8*-mediated chemotaxis (Egholm et al., 2019; Impellizzieri et al., 2019), therefore, the afore-mentioned negative correlations support the concept about exercise-induced NETs formation.

### 4.1 Plausible mechanism of negative correlations between plasma pre-exercise IL-4 and Δ-rLBCL

Interleukin 4 (IL-4) is an important regulator of humoral and adaptive immunity. Numerous cells have receptors for IL-4 and can be activated, stimulated for proliferation or differentiation (e.g., B cells, T cells), by this cytokine. There are also receptors for IL-4 on the plasma membrane of human neutrophils of healthy subjects (Impellizzieri et al., 2019). The binding of IL-4 to these receptors induces their dimerization, activation of receptor-associated Janus kinases, and then leads to the phosphorylation and activation of signal transducer and activator of transcription proteins (Egholm et al., 2019). These phosphorylated proteins undergo dimerization and, after translocation to the nucleus, initiate transcription of target genes. In general, the effect of IL-4 on human PMNs is inhibitory, being one of the mechanisms protecting tissues from excessive damage in the course of immunological response (Egholm et al., 2019; Impellizzieri et al., 2019; Heeb et al., 2020). Preincubation of human PMNs with IL-4 at concentration of 150 ng/mL for 6 h inhibited NETs formation induced by 100 nmoles/L PMA (Impellizzieri et al., 2019). Moreover, these so treated PMNs had lower expression of chemokine receptors (CXCR1, CXCR2) and revealed lower chemotactic response to chemokine CXCL8 (Impellizzieri et al., 2019). Although the IL-4 concentration used in the afore-mentioned experiment was many times higher than that present in the serum of healthy subjects (Kleiner et al., 2013), this cannot exclude the possibility of such an effect of IL-4 *in vivo*. PMA is the strongest and most effective inducer of NETs formation (Hoppenbrouwers et al., 2017), but it is not a physiological activator. Isolation of PMNs can alter their responsiveness to various mediators including IL-4, and so high concentrations must be used in in vitro experiments. However, to our knowledge, no studies focused on NETs inhibition by various IL-4 concentrations have been executed so far. *In vivo*, circulating neutrophils are continuously exposed to IL-4, thus lower concentrations may evoke the inhibitory effect. It is supposed that strenuous exercise induces NETs formation via shear and heat stress (Beiter et al., 2015). NETs formation involves activation of NADPH oxidase, mitochondrial respiratory chain, and increased ROS production (Quindry et al., 2003) that are postulated to be responsible for the increase in rLBCL just after exercise (Chmielecki et al., 2022). Therefore, the inhibitory effect of IL-4 on NETs formation seems to be the most probable explanation of the negative correlation between pre-exercise IL-4 and just-after-exercise ΔrLBCL.

### 4.2 Association of pre-exercise concentrations of other cytokines with rLBCL increment just after exercise

IL-8 and TNF-α can prime PMNs for enhanced ROS production in response to various agonists, including platelet activating factor (PAF), n-formyl-L-methionyl-L-leucyl-L-phenylalanine (fMLP), complement component 5a (C5a) (You et al., 1991; Baggiolini and Clark-Lewis, 1992; Bajaj et al., 1992; Wozniak et al., 1993; Lloyds et al., 1995; Gougerot-Podicalo et al., 1996; Decleva et al., 2002; Guichard et al., 2005). Moreover, 2 h incubation of PMNs from healthy subjects with IL-8 and TNF-α at concentrations of 100 ng/mL and 8 ng/mL, respectively, induced NETs formation, which was accompanied by increased ROS production *in vitro* (Keshari et al., 2012). Therefore, we supposed that baseline circulating levels of IL-8 or TNF-α can affect the post-exercise increase in ROS production by PMNs. Surprisingly, there were no association between pre-exercise and just-after-exercise plasma concentrations of these cytokines and ΔrLBCL just after exercise. The following reasons may explain these negative results: (A)—concentrations of IL-8 and TNF-α used for induction of NETs formation were many times-higher than those occurred *in vivo* in healthy subjects (Kleiner et al., 2013); (B)—increase in plasma cell-free nuclear DNA reflecting NETs formation was observed within several minutes from the onset of strenuous exercise (Breitbach et al., 2014), and, therefore, initial signals switching on this process have rather physical (shear stress related to increased cardiac output and blood flow, and perhaps an increase in body temperature) but not inflammatory nature.

Jogging on a treadmill at 70% of maximum heart rate for 15 min resulted in an increase in the core temperature of the triceps surae to 39°C (Wirth et al., 1998). Preincubation of isolated human PMNs for 1 h at 39.5°C accelerated PMA- and *Pseudomonas* aeruginosa-induced NETs formation (Keitelman et al., 2019). In a working skeletal muscle, alternating blood vessels compression and dilation result in large changes of intramuscular blood flow velocity (Osada et al., 2015) and perhaps shear stress. More, elevated shear stress or turbulent blood flow can promote interaction of platelets with PMNs involving von Willebrand factor and binding of platelet αIIbβ3 to SLC44A2 on PMNs promoting mechanosensitive-dependent formation of NETs (Constantinescu-Bercu et al., 2020). On the other hand, in an *in vitro* microfluidic model of sterile thrombotic occlusion, increased rheological forces triggered NETs formation that was insensitive to inhibitors of platelet-PMNs adhesion (Yu et al., 2018). This may also explain the weak negative association between pre-exercise concentration of anti-inflammatory IL-10 and ΔrLBCL just after exercise.

### 4.3 Associations of plasma concentrations of monitored cytokines with rLBCL decrement at 1, 3, and 24 h post-exercise

The increase in rLBCL noted just after exercise was transient and from 1 h post-exercise till the end of observations at 24 h post-exercise, a significant decrease in rLBCL was noted in comparison to pre-exercise value. Since IL-8 and TNF-α can prime neutrophils for enhanced ROS production (You et al., 1991; Baggiolini and Clark-Lewis, 1992; Bajaj et al., 1992; Wozniak et al., 1993; Lloyds et al., 1995; Gougerot-Podicalo et al., 1996; Decleva et al., 2002; Guichard et al., 2005), we expected that subjects with higher plasma concentrations of these cytokines would have smaller post-exercise decrements in rLBCL (Δ-rLBCL). However, the Spearman’s *ρ* analysis did not reveal such associations. These negative results can be explained by (A)—a transient nature of post-exercise increase in plasma IL-8 and TNF-α; (B)—*in vivo* concentrations of these cytokines are substantially lower then their concentration that evoked priming effect on PMNs *in vitro* (You et al., 1991; Baggiolini and Clark-Lewis, 1992; Bajaj et al., 1992; Wozniak et al., 1993; Lloyds et al., 1995; Gougerot-Podicalo et al., 1996; Decleva et al., 2002; Guichard et al., 2005); (C)—there are numerous other substances that can *in vivo* counterbalance these two pro-inflammatory cytokines: for instance, circulating catecholamines that rose during exhaustive exercise were reported to inhibit NETs formation (Vogelgesang et al., 2017); (D)—at 3 h post-exercise the blood phagocyte count increased by about 3-times (Chmielecki et al., 2022), indicating that a new population of these cells from marginated pool and bone marrow entered the circulation with probably different responsiveness to IL-8 and TNF-α in comparison to pre-exercise cells.

Exercise caused the highest increase in IL-10 that was noted just after and at 1 h post-exercise. This cytokine has anti-inflammatory activity and inhibits ROS production from human PMNs. Thus, one may expect that sportsmen presenting higher increase in IL-10 would have greater decline in rLBCL at 1 h and later after exercise. Similarly, no such associations were found. This is in agreement with our previous suggestion that post-exercise decrease in rLBCL may result from an increased number of younger PMNs from bone marrow that have lower ability to produce ROS (Chmielecki et al., 2022). It should be noted that Spearman analysis of IL-4 plasma levels with rLBCL decrements always revealed negative value of *ρ*, and for pre- and just-after-exercise IL-4 and ΔrLBCL at 1 h post-exercise, the negative correlations reached borderline significance, in means that sportsmen with higher pre-exercise concentrations of IL-4 would have a deeper decrement of rLBCL at 1 h post-exercise (Figure 9). In an animal model of antibody-induced arthritis, IL-4 suppressed the egress of PMNs from bone marrow and their migration to inflamed joints (Panda et al., 2020). Assuming that IL-4 can inhibit exercise-induced exit of young PMNs from bone marrow in humans, this action could have a completely opposite effect: higher pre-exercise IL-4 would cause a lower post-exercise increment in the number of young immature circulating PMNs and a lower decrement of rLBCL at 1 h post-exercise. To check this hypothesis, we additionally analyzed associations between pre-exercise plasma concentrations of IL-4 and exercise-induced increment in the number of circulating phagocytes just after, and at 1, 3, and 5 h post-exercise. At any time point, no significant associations were found (Spearman’s *ρ* ranged from −0.04 to −0.26, *p* > 0.3). Therefore, the plasma IL-4 effect on exercise-induced movement of blood phagocytes (namely, PMNs) from bone marrow into the bloodstream seems less likely. On the other hand, addition of IL-4 to suspensions of isolated human PMNs preactivated with INF-γ or TNF-α resulted in inhibition of hexose monophosphate shunt, which is involved in ROS production (Bober et al., 2000). It is possible that post-exercise PMNs, conditioned by various mediators released in response to exercise, could be inhibited by circulating IL-4, explaining the afore-mentioned negative correlation between IL-4 and rLBCL decrement at 1 h post-exercise.

## 5 Conclusion

To the best of our knowledge, this is the first study conducted in such strictly monitored conditions which includes kinetics and control (reference day). Furthermore, the observation and correlation of selected cytokins have not been performed before. This article may be of interest to a physiologist, sports coaches, but also for scientists who wonder about the relationship between exercise and physiological stress.”

We found that exhaustive exercise caused transient increase in plasma concentration of IL-8, IL-10 and TNF-α in healthy amateur sportsmen. Although these cytokines can affect the activity of PMNs, no association of these cytokine plasma levels (pre- and post-exercise) with biphasic changes of rLBCL (increase just after exercise and decrease at subsequent time-points), reflecting ROS production by blood phagocytes, was noted. Plasma concentrations of IL-4 were not altered by the exhaustive treadmill run, however pre- and just-after-exercise concentrations of this cytokine were negatively associated with the increment in rLBCl just after exercise. Moreover, pre- and just-after- exercise plasma IL-4 levels tended to negatively correlate (borderline significance) with the decrease of rLBCL at 1 h post-exercise. This suggests that IL-4 can prevent excessive ROS production by blood phagocytes just after an exhaustive exercise and augment the second phase consisting in suppression of oxidants generation post-exercise. Therefore, circulating IL-4 can be involved in maintaining a proper balance between oxidants and antioxidants during strenuous exercise and post-exercise recovery. However, confirmation of this hypothesis requires further studies. In conclusion, despite the observed increment in some pro-inflammatory cytokines, the rise was transient and compensated by the increase in anti-inflammatory cytokines. Therefore, it seems that exercise, even to exhaustion, is relatively safe for people who practice sport.

## Data Availability

The raw data supporting the conclusion of this article will be made available by the authors, without undue reservation.

## Abbreviations

IL-4, interleukin 4; IL-6, interleukin 6; IL-8, interleukin 8; IL-10, interleukin 10; TNF-α, tumor necrosis factor alpha; VO_2_, max maximal oxygen consumption; LBCL, luminol-enhanced whole blood chemiluminescence; a-rLBCL, absolute resting (spontaneous) LBCL; rLBCL, resting (spontaneous) LBCL normalized per phagocyte count; PMNs, polymorphonuclear leukocytes; ROS, reactive oxygen species; NETs, neutrophil extracellular traps; cf-nDNA, circulating cell free nuclear DNA; PMA, phorbol 12-myristate 13-acetate; RLU, relative light units; CK, creatine kinase.
